# The fluid factor OVGP1 provides a significant oviductal microenvironment for the reproductive process in golden hamster^†^

**DOI:** 10.1093/biolre/ioad159

**Published:** 2023-11-23

**Authors:** Kenji Yamatoya, Masaru Kurosawa, Michiko Hirose, Yoshiki Miura, Hikari Taka, Tomoyuki Nakano, Akiko Hasegawa, Kyosuke Kagami, Hiroshi Yoshitake, Kaoru Goto, Takashi Ueno, Hiroshi Fujiwara, Yoichi Shinkai, Frederick W K Kan, Atsuo Ogura, Yoshihiko Araki

**Affiliations:** Institute for Environmental & Gender-Specific Medicine, Juntendo University Graduate School of Medicine, Chiba, Japan; Institute for Environmental & Gender-Specific Medicine, Juntendo University Graduate School of Medicine, Chiba, Japan; Bioresource Engineering Division, RIKEN BioResource Research Center, Ibaraki, Japan; Laboratory of Proteomics & Biomolecular Sciences, Biomedical Research Core Facilities, Juntendo University Graduate School of Medicine, Tokyo, Japan; Laboratory of Proteomics & Biomolecular Sciences, Biomedical Research Core Facilities, Juntendo University Graduate School of Medicine, Tokyo, Japan; Department of Anatomy and Cell Biology, Yamagata University School of Medicine, Yamagata, Japan; Department of Obstetrics & Gynecology, Hyogo Medical University, Hyogo, Japan; Department of Obstetrics & Gynecology, Kanazawa University Graduate School of Medical Sciences, Ishikawa, Japan; Institute for Environmental & Gender-Specific Medicine, Juntendo University Graduate School of Medicine, Chiba, Japan; Department of Anatomy and Cell Biology, Yamagata University School of Medicine, Yamagata, Japan; Laboratory of Proteomics & Biomolecular Sciences, Biomedical Research Core Facilities, Juntendo University Graduate School of Medicine, Tokyo, Japan; Department of Obstetrics & Gynecology, Kanazawa University Graduate School of Medical Sciences, Ishikawa, Japan; Cellular Memory Laboratory, RIKEN Cluster for Pioneering Research, RIKEN, Saitama, Japan; Department of Biomedical and Molecular Sciences, Faculty of Health Sciences, Queen’s University, Kingston, ON, Canada; Bioresource Engineering Division, RIKEN BioResource Research Center, Ibaraki, Japan; Institute for Environmental & Gender-Specific Medicine, Juntendo University Graduate School of Medicine, Chiba, Japan; Division of Microbiology and Immunology, Department of Pathology and Microbiology, Nihon University School of Medicine, Tokyo, Japan; Department of Obstetrics & Gynecology, Juntendo University Graduate School of Medicine, Tokyo, Japan

**Keywords:** oviductal glycoprotein 1 (OVGP1), knockout-hamster, infertility, early developmental failure, embryonic lethality

## Abstract

The mammalian oviductal lumen is a specialized chamber that provides an environment that strictly regulates fertilization and early embryogenesis, but the regulatory mechanisms to gametes and zygotes are unclear. We evaluated the oviductal regulation of early embryonic development using *Ovgp1* (encoding an oviductal humoral factor, OVGP1)-knockout golden hamsters. The experimental results revealed the following: (1) female *Ovgp1-*knockout hamsters failed to produce litters; (2) in the oviducts of *Ovgp1*-knockout animals, fertilized eggs were sometimes identified, but their morphology showed abnormal features; (3) the number of implantations in the *Ovgp1*-knockout females was low; (4) even if implantations occurred, the embryos developed abnormally and eventually died; and (5) *Ovgp1-*knockout female ovaries transferred to wild-type females resulted in the production of *Ovgp1*-knockout egg-derived OVGP1-null litters, but the reverse experiment did not. These results suggest that OVGP1-mediated physiological events are crucial for reproductive process in vivo, from fertilization to early embryonic development. This animal model shows that the fate of the zygote is determined not only genetically, but also by the surrounding oviductal microenvironment.

## Teaser

Deficiency of an oviductal humoral factor (OVGP1) caused female infertility in golden hamsters. The presence or absence of OVGP1 has significant physiological effects on early embryonic development in vivo.

## Graphical Abstract

**Graphical Abstract:**
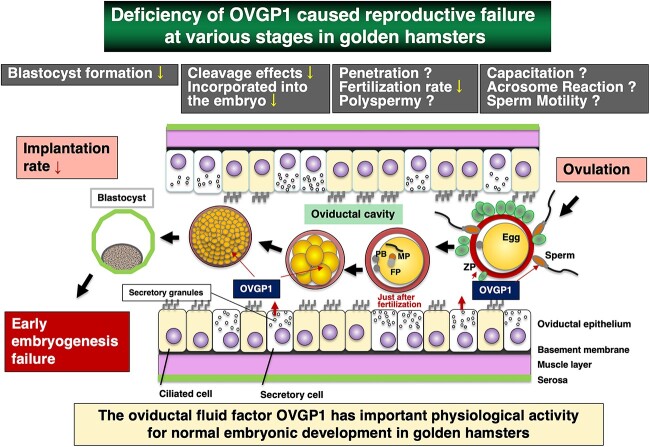

## Introduction

The use of culture medium with a well-defined composition in mammalian in vitro fertilization (IVF) was pioneered by Yanagimachi and Chang using the golden hamster (*Mesocricetus auratus*) as an animal model [1]. The theory subsequently developed into a fundamental principle for the development of human IVF-embryo transfer (ET) methods [2]. IVF-ET is performed worldwide as a treatment for infertility, especially in human. Oocytes are harvested directly from the ovaries without passing through the fallopian tubes (oviducts), fertilized, and cultured in test tubes (dishes), then transferred vaginally into the uterine cavity. Therefore, oviductal factors are not always necessary in human IVF-ET, and few studies have focused on the reproductive physiology of the oviducts, especially during the last two decades. However, the oviduct (or its homologous organ), the original site of fertilization and early embryogenesis in sexual reproduction, is widely conserved in both lower vertebrates and mammals [3–5]. Since the origin of sexually reproducing organisms can be traced back to the Cambrian period, at least 600 million years ago (for reviews, see [6, 7]), the oviduct may have important as-yet-unknown functions in the process.

The association of cyclic fluctuations in sex hormones with the morphology and function of the oviduct highlights the need for detailed studies of the reproductive physiology of this organ [8, 9]. The medium for IVF-ET has a long history of development based on the composition of the Fallopian tube and uterine fluids [10–12], including the concentrations of glucose, inorganic salts, growth factors, and hormones; however, at the time those cultures were developed, the proteins (i.e., the main components of oviductal fluid) were largely unknown. Therefore, serum components were used as a substitute for the other components of the oviductal fluid. However, the goal of IVF should be to reproduce the in vivo oviductal microenvironment as closely as possible, as a medical treatment. It is also biologically important to elucidate the reproductive physiology of the oviduct, or the site of fertilization and early embryonic development in most sexual reproductive species, including mammals.

In this study, using the golden hamster, we demonstrate that an estrogen-dependent humoral factor in the oviduct, oviductal glycoprotein 1 (OVGP1), has a significant effect on reproductive process in vivo*.*

## Materials and methods

### Animals and analysis of the genotype

Sexually mature (7–8-week-old) golden hamsters (*M. auratus*) were purchased from Japan SLC, Inc. (Hamamatsu, Shizuoka, Japan). They were maintained and bred in our animal facilities under 12L:12D conditions. All animal experiments were performed in accordance with the guidelines for the care and use of laboratory animals, Juntendo University (approval # 768) and RIKEN Tsukuba Institute (approval # T2021-Jitsu004), Japan. For genotyping, ear biopsies were lysed with 0.4 mg/mL Proteinase K (Nakalai Tesque Inc., Kyoto, Japan) and partially purified by standard chloroform extraction. Genomic fragments containing the target site were amplified by PCR using primers (forward 1: 5'-AAGCCAGAATCCAAAGCTGAAGCAC-3′; reverse 1: 5'-GTATTAAACCCTCACAACTGGGCTC-3′; the expected product length of wild-type (WT)-allele: 1888 bp and *Ovgp1*-knockout (KO)-allele: −300 bp). The PCR procedure followed the instructions for Tks Gflex DNA polymerase (TaKaRa Bio Inc., Shiga, Japan) as follows: Genomic DNA of each hamster ear punch was amplified in 1x Gflex PCR buffer (containing 1 mM MgCl2, 200 μM dNTP plus), Tks Gflex DNA polymerase (0.625 units) and 0.4 μM of the primers in a total volume of 25 μL. The samples were incubated at 94°C for 1 min, then subjected to 35 cycles of denaturation at 94°C for 10 s, annealing at 60°C for 15 s and extension at 68°C for 20 s using a programmable thermal controller. Upon completion of the reaction, the sample was incubated at 72°C for 1 min and stored at 4°C until use. The WT allele was also confirmed with a primer set (forward 2: 5'-CGCTGGGCCACTTGCTGTTTG-3′; reverse 1: (see above)) designed to amplify only the WT allele (using this primer set, only the WT allele produces a PCR product of 756 bp), just in case for strain maintenance (data not shown). The amplified PCR fragments were subcloned into the pGEM T vector system (Promega Corporation, Madison, WI, USA) and sequenced to confirm each allele.

### Generation of *Ovgp1*-KO hamsters

**Figure 1:**
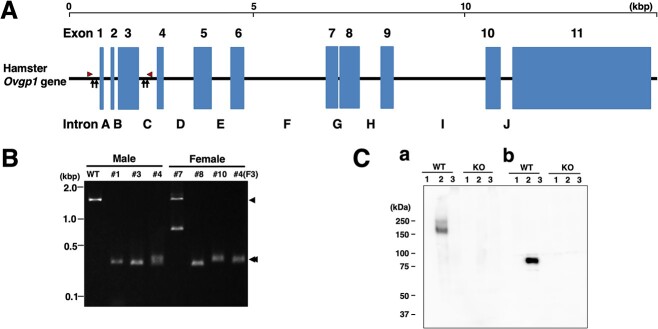
*Production of Ovgp1-null hamsters.* Gene structure of hamster *Ovgp1* (from GCF_017639785.1) and its editing strategy (A). The position of the gene sequence to be removed from exon 1 to 3 is indicated by vertical arrows and the position of PCR primers for mutant detection is indicated by arrowheads (for details, see the Materials and Methods section). Genotypes of F0 animals after gene editing by PCR (B). The positions of the predicted PCR products relative to the genomic DNA are indicated by arrowheads (WT) and double arrowheads (KO), respectively. Male #1, 3, 4 and female #8, 10 were successfully gene edited as designed. Male #1, female #8, #10 did not produce pups, so males #3 and #4 were used to maintain the strain. #4(F3) indicates that DNA extracted from an F3 generation female derived from a #4 F0 male individual was used as the template. Western blotting analysis using OVGP1-specific Abs (C); equal amounts of tissue protein solution (a same lot of protein solution that was adjusted at the same time) were detected by SDS-PAGE followed by OVGP1-specific Abs (AZPO8, recognizing carbohydrate moiety of the OVGP1 (a); ab74544, recognizing the N-terminal peptide of OVGP1 (b)). Lanes 1: ovary, 2: oviduct, and 3: uterus, respectively.

*Ovgp1*-KO hamsters were generated using an in vivo electroporation CRISPR-Cas9 system, essentially as described previously [13]. Pairs of sgRNAs were designed to delete the *Ovgp1* genomic sequence from exons 1 to 3 (sequence of DNA targets: + allele; 5′-ACTGACTCCCTGCTAGCGTCAGG-3′; 5′-CCTGCTAGCGTCAGGCCACGGAT-3′: - allele; 5′-CCATCGACCAGCCCCCTGAGCTG-3′; 5′- CCTCGATGACTTGGGAGTTAATG-3′) (Figure 1A). Ten animals were born of which three males (#1, 3, 4) and two females (#8, 10) appeared to be homozygous defects in the target gene region (Figure 1B). Females #8 and #10 did not show any external signs of pregnancy in mating experiments with WT males with confirmed fertility; two males (#3, 4) were fertile and the defective gene was transmitted to their offspring when mated with WT females. To minimize the potential effects of off-targeting mating of heterozygotes, two generations of heterozygotes were mated to WT and the heterozygotes were mated to homozygous males derived from #3 and #4, respectively. The sequences of the gene editing sites in the KO allele were confirmed by direct sequencing analysis. These heterozygous animals were found to produce a normal number of offspring.

### Western blot analysis

The concentration of total protein extracted from animal organs was quantified using BradfordUltra (Expedeon Ltd, Cambridgeshire, UK). The following antibodies (Abs) specific for hamster OVGP1 used in this study: AZPO-8 (monoclonal Ab (mAb) against the oligosaccharide portion of hamster OVGP1 (mouse IgG1) [14]); anti-OVGP1 N-terminal-peptide polyclonal Ab (pAb) (rabbit IgG, ab74544; Abcam plc, Cambridge, UK); horse radish peroxidase (HRP)-conjugated anti-mouse IgG pAb (P0260); and anti-rabbit IgG pAb (P0448) (Dako, Carpinteria, CA, USA). Equal amounts of proteins were separated by SDS-PAGE system and transferred to Immobilon-P membrane (Merck KGaA, Darmstadt, Germany). Immunoreactions were detected according to standard methods described previously [15, 16].

### Collection of eggs

Eggs were collected from the oviducts of mature females by natural mating with fertile males. Coitus was confirmed by the presence of vaginal spermatozoa in the post-ovulatory vaginal discharge and this day was defined as 0-day post coitus (dpc). In particular, fertilization at the 1-cell stage was assessed according to the formation of the female and male pronuclei, a fertilizing sperm tail in the cytoplasm and a second polar body, as described previously [17–19].

### Morphologic observation

Animal tissues were fixed in 20% formalin solution (FUJUFILM Wako Chemical Co., Osaka, Japan) and embedded in paraffin wax according to standard procedures. Three-μm thick sections were cut and stained with hematoxylin–eosin for light microscopy.

To detect the implantation site(s), a solution of Chicago Sky Blue 6B (Tokyo Chemical Industry Co., Ltd, Tokyo, Japan) diluted to 1% in PB saline (PBS) was injected into the left ventricle of female animals anesthetized with sevoflurane (Maruishi Pharmaceutical Co., Ltd, Osaka, Japan). Ten minutes after injection, the blood was perfused with 50 mL of PBS to clearly visualize the blue dye pigment deposited in the uterus.

### Ovarian transplantation

The technique of ovarian transplantation used in this study essentially followed the methods reported elsewhere [20–22]. Briefly, ovaries were isolated from donor animals (5–14-week-old), and suspended in PBS on ice. Ovaries were divided into two equal parts with scissors after removing excess surrounding tissue. Donor animals were anesthetized with sevoflurane, then the ovaries are pulled out of the body through a dorsal approach, and a small incision was made in the ovarian sac under a stereomicroscope to remove the ovary mechanically as much as possible. After removal of the ovary, a donor ovarian fragment was placed in the ovarian sac and returned to the abdominal cavity and the skin incision wound was sutured. Recovery was allowed for at least 2 weeks after surgery, and each individual was then used for mating experiments.

### Statistical analysis

Early embryo morphology of WT and KO animals at 2.5-dpc in the oviduct was classified into three groups as described above, and the number of early embryos in each group of WT and Ovgp1 KO was analyzed using the Mann–Whitney *U* test. The number of implantations at 5.5-dpc of WT and *Ovgp1*-KO females and the number of litters between ovarian implantation groups were also tested in the same way. Fisher exact test was used to compare the fertility of *Ovgp1*-KO and heterozygous female and *Ovgp1*-KO male pairs. A probability of *P* < 0.05 was considered statistically significant.

## Results

### Generation of OVGP1-deficient hamsters

Among oviductal factors in oviductal fluid, OVGP1 is implicated in the reproductive process of several mammalian species, including humans (for review, see [23–27]). Using the golden hamster as a model, which has provided a wealth of knowledge concerning mammalian reproductive processes [28], *Ovgp1*-knockout (KO) animals were generated via gene editing (Figure 1A and B). Preliminary mating experiments showed that the F0 *Ovgp1*-KO females (two independent individuals; #8 and #10) with WT male animals (confirmed fertile) did not produce offspring for more than 15 weeks. By contrast, *Ovgp1*-KO males (#3 and #4) were found to be as fertile as those of the WT. In the F1 and later generations, fertility was confirmed in 17 of 23 (73%) *Ovgp1*-KO males. When WT females were mated with *Ovgp1*-KO males to confirm their fertility, 8 of the 10 pairs (80%) of females gave birth, with an average litter size of 8.00. Fertility was also confirmed to be possible in heterozygous females; 28 of 45 (62%) heterozygous female individuals in the post-F1 generation were confirmed to be fertile, with an average litter size of 7.08. Although fertility in heterozygous females tended to be lower than in the WT fertility rate, there was no statistically significance, as determined by Fisher exact test (*P* = 0.47). Therefore, we maintained a line of *Ovgp1*-KO males and heterozygous females for further experiments.

Western blotting with OVGP1-specific Abs (AZPO8: reactive with the oligosaccharide moiety of OVGP1 [14]; ab74544 (Abcam): reactive with the N-terminal peptide of OVGP1) did not detect OVGP1 in *Ovgp1*-KO hamster oviducts (Figure 1C). In hamsters, OVGP1 is detected in a broad band by AZPO8 with a molecular mass of 200 kDa due to its different degrees of glycosylation [14], and the molecular mass of the core protein of ~70 kDa [29]. The ab74544 antibody would be difficult to detect by Western blotting in the presence of highly glycosylated forms of OVGP1. In mating experiments using F0-F2 mature *Ovgp1*-KO females with WT males with confirmed fertility (total of 15 pairs), no litter was obtained from *Ovgp1*-KO females (Supplementary Figure S1A). These results suggest a lack of fertility in *Ovgp1*-KO females. However, an F0 female (#10) did not show outward signs of pregnancy but went into shock and died suddenly at 15-dpc (almost full-term) after several mating sessions (Supplementary Figure S1B). Since this phenomenon was limited to this case (one F0 individual), we could not conclude that it was the result of *Ovgp1* gene editing. However, this may present an example of the impacts of OVGP1 on the reproductive process in the hamster models.

### Fertilization of *Ovgp1*-KO females

To assess the reproductive process, we examined the development of eggs after mating. In a preliminary experiment, some 1-dpc eggs were fertilized (based on the observation of male/female pronuclei and sperm tail in the egg cytoplasm) in *Ovgp1*-KO animals, but obviously fewer than in the WT (Supplementary Figure S2A (control), D). When examined by light microscopy (binary image), the egg cytoplasm of *Ovgp1*-KO hamsters showed a central accumulation of intracellular organelles (Supplementary Figure S2B (control), E). Although the differences between unfixed eggs from WT and *Ovgp1*-KO hamsters were not always clear under light microscopy, electron microscopy showed that the 1-dpc *Ovgp1*-KO eggs had a thinner zona pellucida (ZP) and a heterogeneous distribution of intracellular organelles after fixation with glutaraldehyde (Supplementary Figure S2C (control), F).

Despite no obvious differences between the ZP of post-ovulatory oocytes in the oviduct and mature ovarian oocytes at the light microscopy level, immunostaining with an OVGP1-specific mAb confirmed the differences in their molecular structures [14]. Previous analysis using Northern blot showed that the expression of *Ovgp1* mRNA in organs other than the oviduct was below the detection level [29]. Therefore, we re-analyzed the expression of *Ovgp1* in oviduct, ovary, and uterus in WT using quantitative RT-PCR. The results showed that the expression of *Ovgp1* in the ovary and uterus was low, even at the RT-PCR level (Supplementary Figure S3). This suggested that OVGP1 contributes little to ovarian ZP formation in hamsters. Accordingly, the thinning of the ZP observed by electron microscopy may have been due to the presence or absence of modification of OVGP1 in the ZP of the oviduct, i.e. differences in the chemical composition of ZP in the oviduct could be visualized as morphological changes by glutaraldehyde fixation.

**Figure 2:**
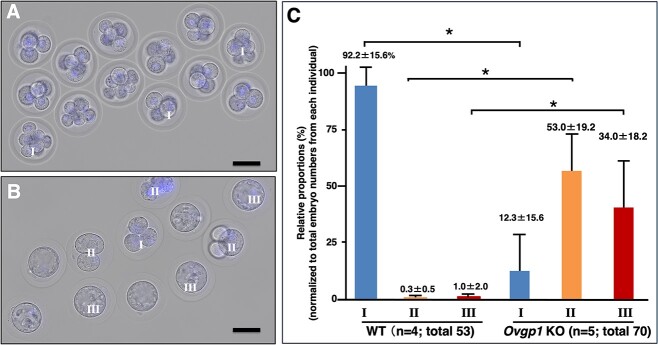
Morphologically observation of early embryos at 2.5-dpc. Coitus with fertile male animal was confirmed as described in the Materials and Methods, early embryos from mature female hamster oviducts were harvested. Typical findings of early embryos from oviduct of WT (A) and *Ovgp1*-KO animals (B), respectively. Each total embryo was morphologically divided into the following three groups: (I) those with normal oocyte division and reaching 4–8-cell stage; (II) those with oocyte division but unevenly divided spheres, those not reaching the 4-cell stage, or their degeneration; and (III) those with no oocyte division. Relative proportions (%) based on total number of embryos from each experiment. The observed numbers of each group were statistically analyzed between group classified in the same group from mature WT and *Ovgp1*-KO females with fertile WT male animals (C). Totally 53 (WT: *n* = 4; 13.25 ± 2.06 (mean ± SD)) and 70 (*Ovgp1* KO: *n* = 5: 14.00 ± 3.08) embryos were examined. During this period, most of the eggs in WT developed into 4–8 cells, while various eggs were observed in KO that was significantly unbalanced or delayed egg division or degenerated (*P* < 0.05: asterisk). Bars = 50 μm.

Next, we morphologically evaluated embryonic development in early oviductal embryos (at 2.5-dpc) from mature (8–15-week-old) WT female hamsters (*n* = 4; total 53 embryos), and from *Ovgp1* KO females (*n* = 5: total 70 embryos). From each individual, 13.25 ± 2.06 (mean ± SD) (WT) and 14.00 ± 3.08 (*Ovgp1*-KO) embryos were recovered. WT embryos at 2.5-dpc typically have advanced oocyte division to the 4–8-cell stage (Figure 2A). Each total embryo was morphologically divided into the following three groups: (I) those with normal oocyte division that had reached 4–8-cell stage; (II) those with oocyte division but unevenly divided spheres, those not reaching the 4-cell stage, or those that had degeneration; and (III) those with no oocyte division (Figure 2A and B). In contrast to the synchronous development of four to eight cells in almost all fertilized eggs in the WT at 2.5-dpc in the oviducts, *Ovgp1*-KO females showed significantly different developmental abnormalities, such as delayed development, disproportionate egg breakage, and degeneration (Figure 2C). At this time point, as with 1-dpc, no clear change in the ZP was observed by light microscopy. As seen in Figure 2C, ~30% of the eggs did not show normal embryonic development, i.e. there was no clear evidence of fertilization (classified as group III). However, the remaining eggs were considered fertilized (I, II) because embryonic development was in progress, although some abnormalities were observed. These results suggested that both fertilization and early development are affected by the loss of OVGP1 in the oviductal microenvironment.

**Figure 3:**
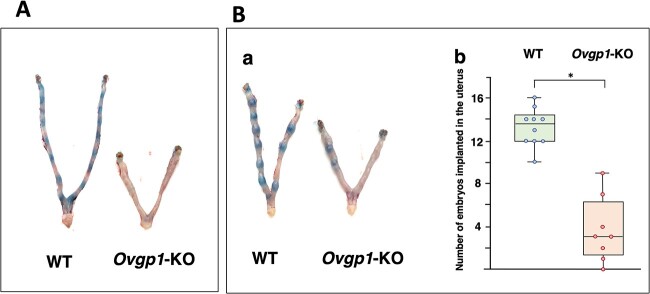
Observation of fetal implantation in the uterus of hamsters*.* At 4.5-dpc (A), and 5.5-dpc (B). Female animals were mated spontaneously with fertile WT males, and showed typical uterine appearance at 4.5-dpc and 5.5-dpc (b). Box-and-whisker diagram of the number of implantations at 5.5-dpc (c). *P*-value was calculated by the Mann–Whitney *U* test. *P < 0.01.

Based on these qualitative findings at the time of fertilization, we examined the events at the early stages of implantation. At 4.5-dpc in WT, implantation sites could be visualized when perfused with Chicago Blue (*n* = 3, all positive staining). However, in a preliminary experiment, no implantation site was observed in *Ovgp1*-KO (Figure 3A). Because the findings in KO individuals were negative, observations at 4.5-dpc were withheld and we speculated that early embryos did not implant in *Ovgp1*-KO females. However, at 5.5-dpc, a few implantation sites were observed in *Ovgp1*-KO hamsters, albeit significantly fewer than in the WT (Figure 3B). These results showed that at least some embryos produced by *Ovgp1-*KO females were able to implant in the uterus.

### Histological findings of 8.5-dpc embryos from *Ovgp1*-KO females

**Figure 4:**
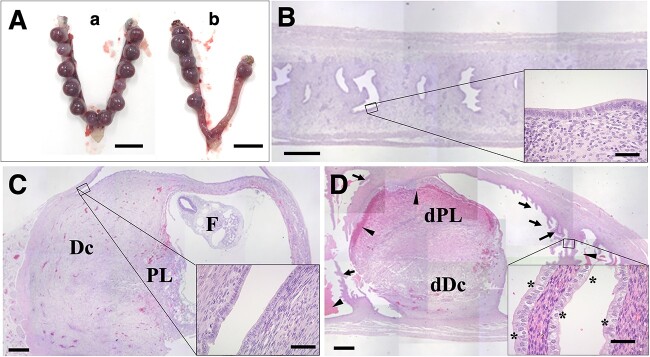
Morphological findings of the uteri at 8.5-dpc in hamsters*.* (A) Appearance of the uteri in pregnancy; WT (a) and *Ovgp1-*KO (b). Bars: 10 mm. (B) Sagittal section of the uterus of a KO hamster in non-pregnant state. The inset shows a magnified view of the endometrium. Bar: 500 μm (inset: 50 μm). Sagittal sections of pregnant uteri and their corresponding magnified images (insets) of WT (C); *Ovgp1*-KO (D). Dc: decidua cells；PL, placenta; F, fetus; dPL, hemorrhagic degenerated placental tissues; dDC, denatured decidualized membrane cells. Arrowheads indicate hemorrhage traces, and arrows reveal endometrial folds not seen in WT. Cuboidal epithelial cells with spherical nuclei and bright cytoplasm are shown by asterisks (inset). Bars: 500 μm (50 μm in insets).

We next focused on the mid-gestation period (~8.5-dpc). WT implanted embryos were well developed and pregnancy was evident (Figure 4A-a). The uterine epithelium of non-pregnant *Ovgp1*-KO hamsters showed no abnormalities under light microscopy (Figure 4B) compared with WT hamsters. At 8.5-dpc in the WT, the embryo was well developed and the developing embryo and placenta were visible in the fetal sac (Figure 4C). The endometrium, except for the implantation sites, was a single layer of columnar epithelium (Figure 4C, inset).

By contrast, 7.5–8.5-dpc embryos from *Ovgp1*-KO females (*n* = 4) were relatively smaller or less distinct in appearance than the WT. Among the mid-gestation *Ovgp1*-KO females, one was nearly as well developed as the WT in terms of appearance (Figure 4A-b), but no developing fetus was observed by light or stereo microscopy. Under light microscopy, placenta/decidua-like primordial tissue with hemorrhagic degeneration was observed, but no trace of embryo buds could be seen, and there was marked hemorrhage in the placenta and uterine cavity (Figure 4D, arrowheads). It should be noted that the endometrium adjacent to the implantation site differed from that of the WT in that it showed the formation of epithelial folds reminiscent of the ampulla of the oviduct (Figure 4D, arrows). High-power magnification of this area revealed numerous cuboidal cells, each with a spherical nucleus and pale cytoplasm (Figure 4D, inset, marked with asterisks). These were likely secretory cells intercalated with non-secretory cells, reflecting the typical columnar epithelium found in WT.

### Validation of phenotypic rescue after ovarian transplantation

**Figure 5:**
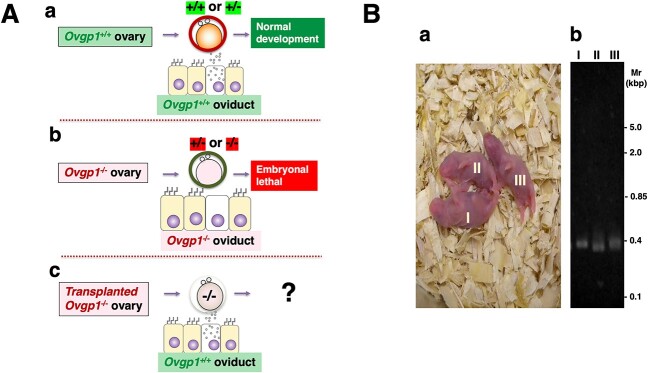
Effects of OVGP1 on early embryogenesis. (A) Schematic of fertilized eggs and oviductal epithelium in early development: Post-fertilization embryos and oviductal epithelium in WT (a) and *Ovgp1*-KO (b) females mated with either WT, heterozygous (*Ovgp1*^+/−^) or KO male, and what happens if a WT female is implanted with an *Ovgp1*-KO ovary and mated with an *Ovgp1*-KO male? (c). (B) Typical results of ovarian transplantation experiments proposed in A-c showing the appearance of litters (a) and their genotyping (b).

Female hamsters whose *Ovgp1* coding region was inactivated by gene editing were defective in fertility and failed to produce litters. This was likely due to the absence of OVGP1 in the oviductal microenvironment of *Ovgp1-*KO female hamsters, which adversely affects early embryonic development immediately after fertilization. This would require to knock-in of the inactivated gene region to determine whether the phenotype was restored, such as in ET experiments. However, hamster eggs (including early embryos) are unfortunately very sensitive to various environmental factors such as light, such that ET experiments with in vitro manipulation pose serious technical difficulties (for review, see Morishita et al. [30]). Thus, instead, phenotype restoration was evaluated using the ovarian transplantation technique (Figure 5).

The fertility of *Ovgp1*-KO male hamsters indicated that the embryo, even if the zygote genotype was *Ovgp1*^+/−^, could develop normally and produce litters in the WT oviduct microenvironment (Figure 5A-a). By contrast, embryos in the oviducts of *Ovgp1*-KO females, whether the oocyte genotype was *Ovgp1*+/− or *Ovgp1*−/−, eventually showed lethal changes due to developmental abnormalities and failed to produce viable offspring (Figure 5A-b).

When these WT females were implanted with KO ovaries and mated with KO males (Figure 5A-c), *Ovgp1*^−/−^ litters (from a total of 26 transplantation experiments, litters were obtained from 11 individuals) were obtained that could not be obtained by normal mating (Table 1; Figure 5B). These litters could only be produced under natural conditions by mating *Ovgp1*^−/−^ males with *Ovgp1*^+/−^ females. Conversely, transplantation of ovaries from WT individuals into KO females did not result in offspring (*n* = 5): preliminary ovarian transplantation experiments using WT females as recipients and donors showed that 11 of 14 (78.6%) transplanted animals produced litters. The possibility that an individual transplanted with ovaries from a WT individual to a KO female could produce litters cannot be ruled out. However, when ovarian transplants were performed with a 78.6% success rate, no litters were produced by any of the five individuals. The probability of such an event occurring is less than 1% (0.04%).

**Table 1:** Fertility of female individuals after ovarian transplantation

Genotype						
Female		Male				
Donor	Recipient		^a^Total experiment number	Number of females that gave birth	Litter size (Mean ± SD)	
WT	WT	WT	17	11^b^	5.22 ± 3.59 (*n* = 9)	
						$ \Big]$ ^c^
*Ovgp1*-KO	WT	*Ovgp1*-KO	27	11^b^	4.83 ± 2.04 (*n* = 6)	
WT	*Ovgp1*-KO	WT	5	0	-	

^a^After ovarian transplantation, the animals were mated with male individuals to check for the presence or absence of pups.

^b^In the 11 deliveries in each group obtained after ovarian transplantation, fetal genotype could not be confirmed due to maternal cannibalism immediately after delivery in two cases (WT to WT transplants) and five cases (*Ovgp1*-KO to WT transplants). In *Ovgp1* to WT transplants, the remaining 6 deliveries of yielded a total of 29 fetuses (mean litter size = 4.83), 19 of which were *Ovgp1*^−/−^. WT to WT transplants produced a total of 47 fetuses (mean litter size = 5.22).

^c^Mann–Whitney *U* test showed no significant difference in litter size between the two transplant groups.

The data presented in Table 1 show the results of ovarian transfer, which enables a more natural and comprehensive assessment of fertilization and implantation than ET. The environment of the oviduct is important for the zygote, and the presence of OVGP1 in the environment surrounding the zygote allows the development of +/− zygotes (zygotes formed by WT males and KO females) and KO zygotes, which normally cannot develop under OVGP-deficient conditions.

## Discussion

We report, for the first time, that deficiency of a fluid factor secreted into the lumen of the oviduct ultimately causes lethal alterations in early embryonic development. Our results are largely phenomenologically consistent with the proposed bioactivity of OVGP1 in various mammalian models, including humans [23–27]. Although OVGP1 has been implicated in sperm function, fertilization, and embryonic development, the final phenotype of KO of this molecule in this study results in lethal alterations in embryonic development after fertilization. Normal embryonic development is a comprehensive process that includes sperm-egg interaction, fertilization, and embryonic development of before and after implantation; therefore, it is premature to conclude from this phenotype that OVGP is essential only for embryonic development. Since the reproductive process from the oviduct to the uterus consists of a series of key events, as described above, each failure is a compound effect of the preceding stages. Interpretation of the phenotype of animals deficient in OVGP1, a molecule originally described in terms of its multiple functions in reproductive physiology, should thus be considered with caution.

In the hamster, OVGP1 has been suggested to be an important fluid factor during in vivo fertilization, especially since it modifies the ZP of oocytes in transit in oviduct after ovulation [14, 31–33]. Inhibition of IVF has also been demonstrated by targeting OVGP1 with a specific antibody [34]. However, the conditions of the in vitro experiments with OVGP1 were different from their in vivo counterparts, which should be kept in mind when interpreting the in vitro data [35]. In addition, OVGP1 is taken up by the developing embryo after fertilization [36], suggesting that it has multiple physiological activities, including early embryonic development and implantation [37]. Our data provide direct evidence of the importance of the physiological function of OVGP1 in the reproductive process of the hamster.

IVF in rodents, including hamsters, is generally performed with oocytes ovulated in the oviduct [38]. This implies that even IVF oocytes are exposed to oviductal factors prior to fertilization. Previous studies have shown that follicular fluid alone is not sufficient to maintain control of oocyte meiosis, which is tightly regulated by the granulosa cell/oocyte syncytium [39]. In addition, hamster IVF oocytes are exceptionally sensitive to the culture environment during the first hours after activation [30]. Thus, the oviducts are thought to play an important role in the early stages of ovulation and subsequent embryonic development [40]. This suggests important physiological activities of the oviduct, especially in vivo. Verification of the relationship between these phenomena and OVGP1 is an important issue that remains to be addressed.

The implications of the lack of a clear phenotype in *Ovgp1*-KO mice described in publication from 20 years ago merit reconsideration; these genetically modified animals produced litters comparable with WT [19]. At present, we cannot elaborate on the phenotypic differences between *Ovgp1*-KO mice and hamsters due to insufficient experimental data. However, the primary structure of the N-terminal region of OVGP1 is highly conserved among species, with considerable interspecies structural diversity in the C-terminus and its degree of glycosylation [23, 27]. Furthermore, until the end of the 20th century, the molecular characterization of OVGP1 was typically performed in large farm animals such as cattle, pigs, and sheep, from which large amounts of oviduct fluid were available, and in baboons as a human model. Studies on the functions of OVGP1 in the reproductive process have mainly used rodents, especially hamsters, which have a stable sexual cycle, but few studies have used mice. There are three reports on mouse OVGP1 from the same research group in the mid-1980s [41–43], but there have been no further reports at the protein level. Despite the major advances in knowledge achieved with the genetic engineering of mouse OVGP1 [44, 45], protein-level studies on the underlying mechanisms that regulate OVGP1 function are lacking. This explains why research in this area has stalled, along with the fact that previous studies of *Ovgp1*-KO mice have not shown a clear reproductive phenotype. In addition, regarding the phenotypic differences between hamsters and mice, we considered the possibility that a functional truncated molecule is formed after KO in mice that serves as a functional replacement, but OVGP1 was not detected by mass spectrometry analysis in mice (Araki, unpublished data). Therefore, we plan to further analyze the phenotypic differences between species of KO animals, including with respect to their chromosomal location of *Ovgp1*, to elucidate the biological activity of this oviduct fluid factor.

Early mammalian development has been studied primarily in mouse models, even before the widespread use of recombinant technology. This is because blastocysts in this species are easier to study using media with a simple chemical composition. The embryos of mice are exceptionally easy to culture embryos in vitro, and the conditions for blastocyst formation were established relatively early in such studies. In hamsters, on the other hand, elucidating the in vitro culture conditions that broke the two-cell block after IVF was much longer process [46]. Although it is now possible to culture hamster cells to blastocysts and obtain litters by ET [47], the unfertilized eggs used in these experiments, whether in hamsters or mice, are collected from oviducts after ovulation. In other words, these eggs are experimentally exposed to oviductal factors prior to the experiment. Therefore, depending on the microenvironment of the oviduct and uterus, the embryos may be subject to a variety of cellular biological control mechanisms that may be important for early embryonic development. This is inferred from the fact that the in vivo interaction between the early embryo and the epithelium of the female reproductive tract is regulated to ensure that the early embryo, an undifferentiated omnipotent cell, has a higher mitotic potential than cancer cells (in humans, a cancer cell rarely grows to the same weight as fetal tissue in the same gestational period) and ensures normal development.

Regarding the molecular expression of Ovgp1 outside the oviduct, the expression of *Ovgp1* in other organs, including ovary and uterus, remains controversial, based on the studies from human, bovine, and other species. Especially in hamsters and mice, pioneering studies using Northern blot analysis showed that the expression of *Ovgp1* in other organs, including female reproductive organs, is negligible compared with that in the oviduct [29, 44]. Furthermore, although faint *Ovgp1* expression was confirmed in the hamster ovary and uterus at the RT-PCR level (Supplementary Figure S3), a relationship between mRNA expression and actual protein translation cannot be concluded without polysome analysis [48]. Therefore, the expression of *Ovgp1* outside the oviduct needs to be more carefully investigated to determine its physiological function(s). Elucidating the molecular mechanisms of the phenotype caused by OVGP1 deficiency is our next research priority.

In conclusion, although IVF-ET, in which ovarian eggs are fertilized in culture medium, transferred vaginally to the uterine cavity, and subsequently implanted, is used as a fertility treatment, the reproductive physiology of the fallopian tubes (oviducts) in humans has long been neglected because of the slight differences in conditions between humans and experimental animals. However, since the oviduct (and its homologous organs), the site of fertilization and early embryogenesis, is widely conserved not only in mammals but also in vertebrates, we speculate that this organ may have important as yet unknown physiological functions (including in the long-term prognosis of embryonic development). From this perspective, the establishment of a hamster model showing a clear phenotype of oviductal factor deficiency is important to assess the physiological significance of the oviduct. The molecular comparison of the reproductive process between *Ovgp1*-KO hamsters in this study and the homologous gene KO mice [19] can be used to investigate the mechanism of mammalian reproduction. Extensive molecular dynamics studies are currently underway using such KO animals. Future research using OVGP1 is likely to address the pathogenesis of infertility due to unexplained early fertilization defects and delayed fetal growth in early pregnancy.

## Supplementary Material

supplementary_figure_1_ioad159

supplementary_figure_2_ioad159

supplementary_figure_3_ioad159

## Data Availability

The data and animals showing in this study will be made available by the corresponding author upon request.
