# Correction to: The fluid factor OVGP1 provides a significant oviductal microenvironment for the reproductive process in golden hamster

**DOI:** 10.1093/biolre/ioae006

**Published:** 2024-01-15

This is a correction to: Kenji Yamatoya, Masaru Kurosawa, Michiko Hirose, Yoshiki Miura, Hikari Taka, Tomoyuki Nakano, Akiko Hasegawa, Kyosuke Kagami, Hiroshi Yoshitake, Kaoru Goto, Takashi Ueno, Hiroshi Fujiwara, Yoichi Shinkai, Frederick W K Kan, Atsuo Ogura, Yoshihiko Araki, The fluid factor OVGP1 provides a significant oviductal microenvironment for the reproductive process in golden hamster, *Biology of Reproduction*, 2023; https://doi.org/10.1093/biolre/ioad159

In the originally published version of this manuscript, the legends to Supplementary Data section Figures 1, 2, and 3 were lacking. The publisher apologises for these lacunae. These three figure files are now emended in the article.

## Supplementary Material

supplementary_figure_1_ioae006

supplementary_figure_2_ioae006

supplementary_figure_3_ioae006

