# Supplementary material for: The fluid factor OVGP1 provides a significant oviductal microenvironment for the reproductive process in golden hamster
Source: Biol Reprod. 2023 Nov 23;110(3):465–75. doi: 10.1093/biolre/ioad159 (PMC10941085; doi:10.1093/biolre/ioad159)
Supplement: supplementary_figure_1_ioad159 [file supplementary_figure_1_ioad159.pdf]

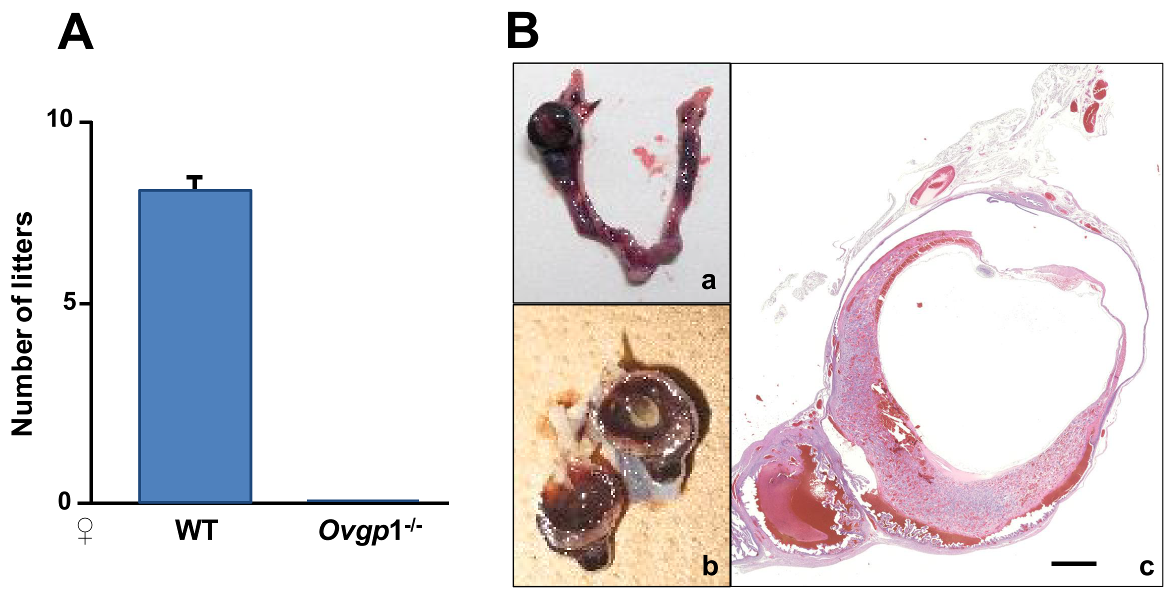

Supplementary Figure S1. **Reproductive ability of *Ovgp1*-KO hamsters.** Fertility of *Ovgp1*-KO female hamsters (A). WT (n=5) and *Ovgp1*-KO (n=15) females mated with fertility confirmed WT males. Autopsy image (B) of an F0 *Ovgp1*-KO female (15-dpc) that died suddenly during a mating experiment: Appearance of the uterus (a), and its cross-sectional image (b). Autopsy revealed that the death had occurred suddenly, because there was a large amount of food in the gastrointestinal tract. Both uterine horns were externally hematomatous, and the split surface had a hematoma visible to the naked eye around the fetal sac, but no fetus was observed. After fixation, light microscopy (hematoxylin-eosin-stained image (c)) revealed a placenta-like structure, but no fetal scar was visible due to absorption and hemorrhage. Bar = 1 mm.
