# Supplementary material for: The fluid factor OVGP1 provides a significant oviductal microenvironment for the reproductive process in golden hamster
Source: Biol Reprod. 2023 Nov 23;110(3):465–75. doi: 10.1093/biolre/ioad159 (PMC10941085; doi:10.1093/biolre/ioad159)
Supplement: supplementary_figure_2_ioad159 [file supplementary_figure_2_ioad159.pdf]

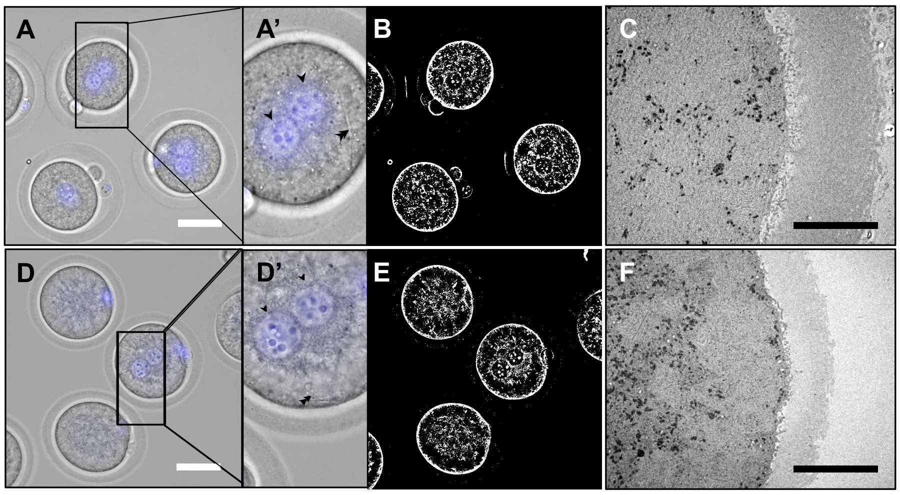

Supplementary Figure S2. Morphological findings of zygotes at 1-dpc in hamsters. Females were spontaneously mated to fertile WT males. Under a dissecting microscope, each oviduct was pricked with a needle and the cumulus mass was excluded as previously reported [20]. The collected zygotes were counterstained with 4',6-diamidino-2-phenylindole solution (FUJI FILM Wako Chemicals) for light microscopic (LM) observation and some were processed for electron microscopy (EM). For transmission EM, samples were fixed with 2.5% glutaraldehyde (FUJIFILM Wako Pure Chemical) in 0.1 M phosphate buffer (PB: pH 7.2) followed by postfixation with 2% OsO_4 in 0.1 M PB. Fixed specimens were dehydrated through a graded series of ethanol, and embedded in Epok812 (Okenshoji Co., Ltd. Tokyo, Japan) according to standard procedure. Images show eggs from WT female (A, B; binary image of A, C; transmission electron microscopy (TEM) image) and *Ovgp1*-KO female (D, E; binary image of D, F; TEM image), respectively. Pronuclei are indicated by arrowheads and sperm tails are shown by double arrowheads in the high magnification images (A', D'). Bars; 50 µm (LM); 10 µm (EM).
