# Supplementary material for: The fluid factor OVGP1 provides a significant oviductal microenvironment for the reproductive process in golden hamster
Source: Biol Reprod. 2023 Nov 23;110(3):465–75. doi: 10.1093/biolre/ioad159 (PMC10941085; doi:10.1093/biolre/ioad159)
Supplement: supplementary_figure_3_ioad159 [file supplementary_figure_3_ioad159.pdf]

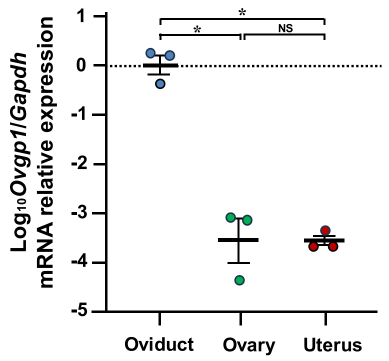

Supplementary Figure S3. Quantitative RT-PCR analysis of reproductive organs of WT hamsters. Ovaries, oviducts and uteri were harvested from sexually mature hamsters (10–11-week-old, n=3) after cervical dislocation under anesthesia and immediately frozen in liquid nitrogen. Total RNA was extracted from each frozen sample using MagNA Lyser (Roche, Basel, Switzerland) and ISOGEN II (NIPPON GENE, Tokyo, Japan). Genomic DNA was then degraded using DNA-free; DNase Treatment and Removal Reagent (Invitrogen, Carlsbad, CA, USA). To synthesize cDNA, 0.4 ug of harvested total RNA was reverse transcribed using PrimeScript RT reagent Kit (Perfect Real Time) (TaKaRa). Relative real-time PCR with the obtained cDNA was performed on a QuantStudio® 5 real-time PCR system (Thermo Fisher Scientific, Waltham, MA, USA) using TB Green® Premix Ex Taq™ II (Tli RNaseH Plus) at 95 °C for 30 s, 40 cycles of 95 °C for 5 s, 60 °C for 30 s. Melting curves were performed to confirm non-specific amplification. The expression level of *Ovgp1* was normalized by a housekeeping gene, *Gapdh*. Primers used in this experiment were: *Ovgp1*; Forward 5'-TGGCTTCCTGGCTTACTATGA-3'; Reverse 5'-TCACGAACATTGCCTTGTAAC-3': *Gapdh*; Forward 5'-CAATGGTGAAGGTCGGAGTGA-3'; Reverse: 5'-CTGGAACATGTAGACCATGTAGT-3'; The relative mean expression of *Ovgp1* in the oviduct, normalized by *Gapdh* (logarithm of the normal), was used as the reference value, and each measurement is expressed as mean±SE. One-way analysis of variance (ANOVA) tests and Tukey's multiple comparison test were used to examine significant differences in *Ovgp1* expression between groups. * p<0.05; NS, not significant
